# Quantitative and semiquantitative estimates of mold exposure in infancy and childhood respiratory health

**DOI:** 10.1097/EE9.0000000000000101

**Published:** 2020-06-19

**Authors:** Jennie Cox, Patrick Ryan, Jeff Burkle, Roman Jandarov, Mark J. Mendell, Gurjit Khurana Hershey, Grace LeMasters, Tiina Reponen

**Affiliations:** a Department of Environmental Health, University of Cincinnati, Cincinnati, Ohio; b Division of Biostatistics and Epidemiology, Department of Pediatrics, Cincinnati Children’s Hospital Medical Center, Cincinnati, Ohio; c Indoor Epidemiology, El Cerrito, California; d Division of Allergy and Immunology, Cincinnati Children’s Hospital Medical Center, Cincinnati, Ohio.

**Keywords:** Asthma, Indoor air, Moisture damage exposure, Mold, Wheeze

## Abstract

Previous epidemiologic studies of dampness and mold relied on metrics that did not fully assess exposure–response relationships. Our objective was to examine quantitative metrics of dampness and mold during infancy and respiratory health outcomes during childhood.

**Methods:**

In-home visits were conducted before age 1 for children in the Cincinnati Childhood Allergy and Air Pollution Study. Respiratory outcomes included age 3 wheeze and age 7 asthma and wheeze. The associations between home exposure and respiratory outcomes were evaluated for 779 children using logistic regression adjusting for household income, neighborhood socioeconomic status, and the presence of pests.

**Results:**

Children residing in homes with ≥0.29 m^2^ of moisture damage were significantly more likely to have wheezing at age 3 and persistent wheeze through age 7 (adjusted odds ratio [aOR] = 2.2; 95% confidence interval [CI] = 1.0, 4.3 and aOR = 3.2; CI = 1.3, 7.5, respectively). Additionally, homes having ≥0.19 m^2^ of mold damage were associated with wheezing at age 3 and early transient wheeze assessed at age 7 (aOR = 2.9; CI = 1.3, 6.4 and aOR = 3.5; CI = 1.5, 8.2, respectively). Mold damage <0.19 m^2^ and moisture damage <0.29 m^2^ were not associated with health outcomes. Mold and moisture damage were also not associated with asthma.

**Conclusion:**

Our data indicate that only the highest categories analyzed for mold (≥0.19 m^2^) and moisture damage (≥0.29 m^2^) in homes at age 1 were significantly associated with wheeze at ages 3 and 7; however, data below these levels were too sparse to assess the shape of the relationship or explore potential health-relevant thresholds.

## Abstract 2

Supplemental Digital Content is available in the text.

**What this study adds**

Methods to quantify the exposure of microorganisms and/or their products are needed to estimate the health risks in damp or moldy buildings so that specific health-relevant guidelines can be set. The goal of this study was to explore measured visible mold and moisture damage to find a quantitative variable which would help determine specific exposure–response relationships regarding dampness and mold and poor respiratory health outcomes. The highest categories analyzed for both variables, moisture damage (≥0.29 m^2^) and mold damage (≥0.19 m^2^), had significant associations with negative health outcomes; however, data below these levels were too sparse to support health-relevant thresholds.

## Introduction

Many studies have explored the role of indoor mold and dampness on adverse respiratory health effects, including asthma development, asthma exacerbation, and respiratory illnesses.^1–4^ To assess the effects of mold and dampness on health, qualitative or quantitative measurements of health effects and of home characteristics, such as measuring visible mold or ascertaining moldy smell, can be made. Qualitative measures of mold and water damage are the most commonly used, as these are the easiest to obtain and can rely on questionnaires. Rydjord et al^5^ found self-reported visible signs of mold or moisture at home during the child’s first year of life were a significant risk factor for ever having wheeze or asthma. A longitudinal study by Jaakkola et al^6^ focused on the effects of parent-reported exposure to molds in dwellings on the development of asthma in childhood. In Jaakkola et al’s^6^ study, living in homes with mold odor at baseline was associated with the development of asthma in the following 6 years, whereas other exposure indicators, such as history of water damage, moisture in the interior surfaces, and visible mold, were not associated with asthma development. Park et al^7^ developed dampness/mold exposure indices for offices, based on questionnaires, which were associated with building-related symptoms reflective of asthma, hypersensitivity pneumonitis, and nasal/sinus disease.

Many epidemiologic studies have used dichotomous metrics of visible mold or of surrogate measures like (1 → 3)-β-D-glucan levels and did not adequately assess dose–response relationships.^8^ Thus, analyses using more specific exposure data are needed for setting protective guidelines. Thresholds of effect for mold exposure need to be further explored. A 90th percentile cut-off value was used in a Boston study, in which exposure to high fungal levels was associated with increased risk of lower respiratory infections in infancy.^9^ In the Boston study, air and dust samples were cultured, individual genera were counted, and high fungal levels were defined as containing at least 1 colony-forming unit (CFU) and being at the 90th percentile within that genus (ranging from 33 to 411 CFU/m^3^ and 400 to 58,000 CFU/g). In a study by Karvonen et al,^10^ trained researchers inspected the child’s main living areas when the child was an average of 5 months old. Any detectable moisture damage with mold was associated with asthma development at age 6.^10^ Research is needed to clarify the dose–response relationships and identify whether or not a possible threshold exists that may that can be easily used in the field and provide helpful information to the public.

Previously, we showed children who resided in homes with more than 0.2 m^2^ of visible mold as an infant were significantly more likely to develop wheezing at 8 months and 3 years than in homes with no visible mold.^11,12^ The objective of this analysis was to conduct a more in-depth evaluation of measured mold and moisture damage to evaluate potential dose–response relationships. Using measured areas of water and mold damage, in addition to qualitative information on moldy odor and history of water damage in the home, we examined specific exposure–response relationships between early-life exposure and childhood wheezing and asthma.

## Methods

### Study population

The Cincinnati Childhood Allergy and Air Pollution Study (CCAAPS) is an ongoing birth cohort in the Greater Cincinnati area. Infants were identified from birth certificate records from October 2001 to July 2003, and eligibility for study enrollment required having a birth record address either <400 m (close) or >1500 m (far) from a major road. In addition, children enrolled in CCAAPS had at least one parent with allergic sensitization confirmed by positive skin prick tests.^13,14^ The study was approved by the University of Cincinnati’s Institutional Review Board, and caregivers provided informed consent before enrollment.

### Health assessments

Children enrolled in CCAAPS completed study visits at ages 1, 2, 3, 4, and 7 including a physical examination, skin prick testing, and parent-completed questionnaires to assess their child’s respiratory health in the previous 12 months. Children completed pulmonary function testing at age 7 as previously described.^15^ In this study, the respiratory health outcomes we considered included recurrent wheezing at age 3, longitudinal wheezing from birth through age 7 (categorized as early transient, late onset, or persistent wheeze), and asthma at age 7. Recurrent wheezing at age 3 was defined by parental report of their child wheezing 2 or more times in the previous 12 months at the age 3 study visit. At age 7, early transient (ET) wheezing was defined as parent-reported wheezing 2 or more times in the previous 12 months at the age 1, 2, 3, or 4 study visit but no wheezing at the age 7 study visit.^15^ Late-onset (LO) wheeze was defined as wheezing 2 or more times in the previous 12 months at the age 7 study visit but no previous wheezing episodes. Persistent (PS) wheezing was defined as parent-reported wheezing 2 or more times in the previous 12 months at the age 1, 2, 3, or 4 study visit and also at age 7. Children with no reported wheezing at any age were categorized as never wheezers. Asthma at age 7 was defined as previously described.^15,16^ Briefly, children were defined as asthmatic if they fulfilled 2 criteria: (1) caregiver report of asthma symptoms (in the previous 12 months, any report of tight chest or throat, difficulty breathing or wheezing after exercise, wheezing and/or whistling in the chest) and (2) demonstration of airway reversibility (defined as ≥12% increase in forced expiratory volume in one second after bronchodilation) or a positive methacholine challenge test result (defined as a ≥20% decrease in baseline forced expiratory volume in one second at a cumulative inhaled methacholine concentration of ≤4 mg/mL).

### Exposure assessments

Each room, including the basement, was visually inspected for signs of mold or water damage, and the location and the size of the damaged surface were recorded on a checklist as exposure at age 1.^11^ Mold damage (m^2^) was defined as the largest measured single surface area with mold, or mold and water damage, in any room in a home (i.e., visible mold was required). Moisture damage (m^2^) was defined as the maximum damaged surface from either water, mold, or both on a single surface in any room in a home. In addition, we also examined participant-reported water damage history yes/no (y/n) and moldy smell detected by a trained researcher (y/n) as previously described.^17^

### Confounders

**Figure 1.**
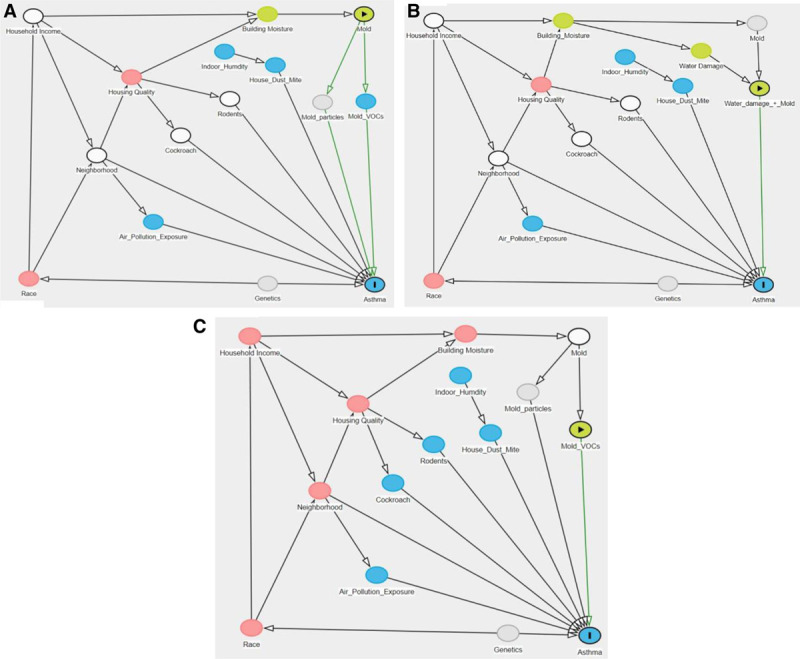
Directed acyclic graph^18^ for the three exposures of interest. Each circle with a dark outline represents potential confounding variables. A green arrow is a causal path of interest for the analysis (e.g., mold in home → asthma). A black arrow means no bias on that path. The pathways in each DAG were the same for the different health outcomes analyzed (age 3 wheeze, wheeze phenotypes assessed at age 7 or age 7 asthma). A, Mold damage as the exposure variable. B, Moisture damage as the exposure variable. C, Moldy odor as the exposure variable.

Directed acyclic graphs (DAGs) were developed for three exposure variables to identify potential confounding variables requiring multivariate adjustment for each outcome analyzed (Figure 1).^18^ A DAG, for causal paths of interest (e.g., mold → asthma), identifies if there are potential biasing paths. Based on the hypothesized connections in the DAG, income, neighborhood socioeconomic status, cockroaches, and rodents were identified as variables requiring adjustment for mold and moisture damage DAGs. The neighborhood socioeconomic status was determined by a deprivation index which utilizes principal components of six different 2015 American Community Survey measures. Rescaling and normalizing forces the index to range from 0 to 1, with a higher index indicating increased community deprivation.^19^ Household income was reported by caregivers at study enrollment and defined as <$29,999, $30,000–$69,999, and ≥$70,000. The presence of cockroaches and rodents (mice and rats) in the home was reported by caregivers at age 1.^20^ For the moldy odor exposure DAG, mold damage was the only confounder variable identified as requiring adjustment.

### Statistical analyses

First, we examined the univariate relationship between each health outcome and exposure and demographic variables, including mold damage, moisture damage, water damage history (y/n), and moldy smell (y/n). A DAGs was constructed for each of the three exposure variables that demonstrated a significant association with a health outcome. Next, we constructed separate logistic regression models, adjusting for variables as identified by the DAGs, for each of the different health outcomes. In addition, we examined mold damage and moisture damage as both continuous and categorical variables. Categorical variables included five categories. Category 0 for the mold and moisture damage variables was homes with no mold damage or no moisture damage, respectively. Categories 1–4 contained homes with mold or moisture damage greater than 0, divided into four quartiles of the nonzero values. In the categorical analyses, each of the exposure quartiles were compared with the reference category of no moisture or mold damage. Evaluating mold and moisture damage categorically was designed to evaluate the odds ratios for the associated outcomes of wheezing and asthma for each category. Models with continuous exposure variables for mold and moisture damage were constructed to estimate the odds ratios associated with each 1 m^2^ increase in the exposure. We also considered moldy odor as the exposure of interest in a separate model, adjusted for visible mold exposure and other factors identified by the DAG. Differences among asthma and wheeze phenotypes at age 7 were tested using Chi-square tests; a *P* value of less than 0.05 was considered statistically significant.

## Results

### Study population and exposure

**Figure 2.**
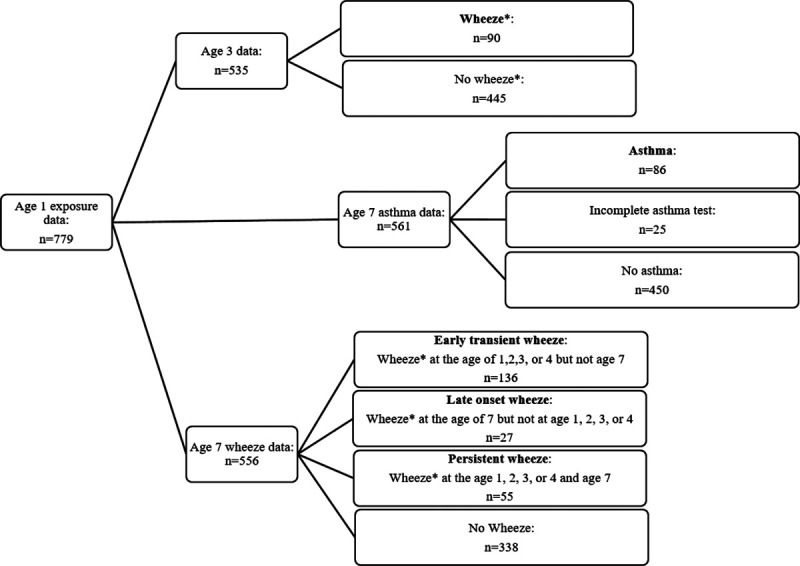
Flowchart of the number of participants with available data and health outcomes. *Wheezing two or more times in the previous 12 months.

**Figure 3.**
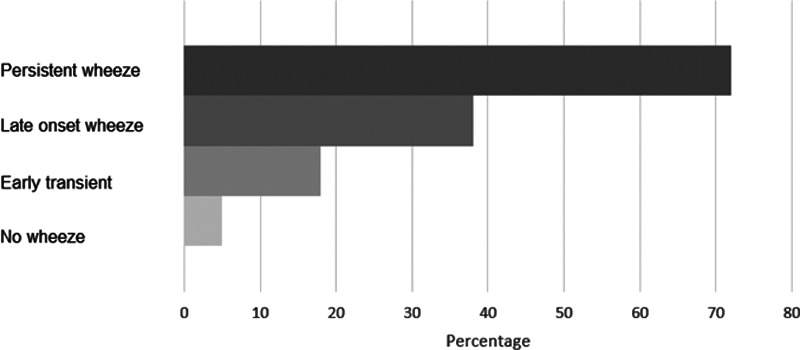
Percentage of age 7 children with asthma by their wheeze phenotype.

Due to some missing values in health outcomes, the number of subjects included in analyses varied for different health outcomes. Of the 535 participants assessed at age 3, 90 had wheeze. Of the 561 participants assessed at age 7, 136 had ET wheeze, 27 had LO wheeze, 55 had PS wheeze, and 86 had asthma (Figure 2 and Table 1). Individuals who had asthma-like symptoms, but were not able to complete either the bronchial hyperresponsiveness pulmonary function test or methacholine challenge (n = 25) were excluded from age 7 asthma analysis. Due to the exclusion of these individuals and to ensure all health outcomes were represented, age 7 asthma and wheeze phenotypes at age 7 were included separately. Asthma, by Chi-square tests (1, N = 536), was significantly correlated with PS wheeze (*P* < 0.001), LO wheeze (*P* < 0.01), and no wheeze (*P* < 0.001), but was not significantly associated with ET wheeze (*P* = 0.50). For those where were able to complete the asthma test, 72% of individuals with PS wheeze also had asthma (Figure 3, and eTable 1; http://links.lww.com/EE/A93).

**Table 1.**
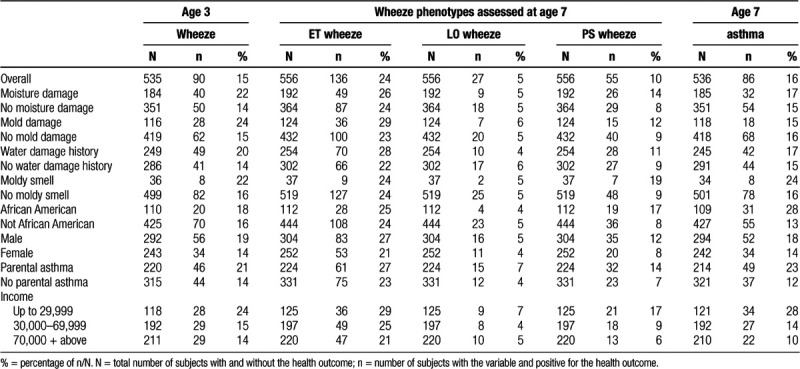
Moisture and mold damage, water damage history, moldy smell, race, gender, parental asthma, and income and the prevalence of age 3 wheeze, wheeze phenotypes assessed at age 7, and age 7 asthma.

A total of 779 participants, with data completed at age 1, had results on mold and moisture damage in their homes. Most homes did not have moisture or mold damage; 35% of homes had moisture damage and 22% had mold damage. As a result, the median, 75%, and maximum values were for mold damage 0, 0, and 4.2 m^2^, and for moisture damage area, 0, 0.0039, and 82 m^2^. The means for mold and moisture damage area were 0.047 and 0.25 m^2^, respectively. Category 1 through 4 minimum values for mold damage were 0.00065, 0.0026, 0.0065, and 0.19 m^2^, and for moisture damage 0.00065, 0.0039, 0.093, and 0.29 m^2^ (Table 2).

**Table 2.**
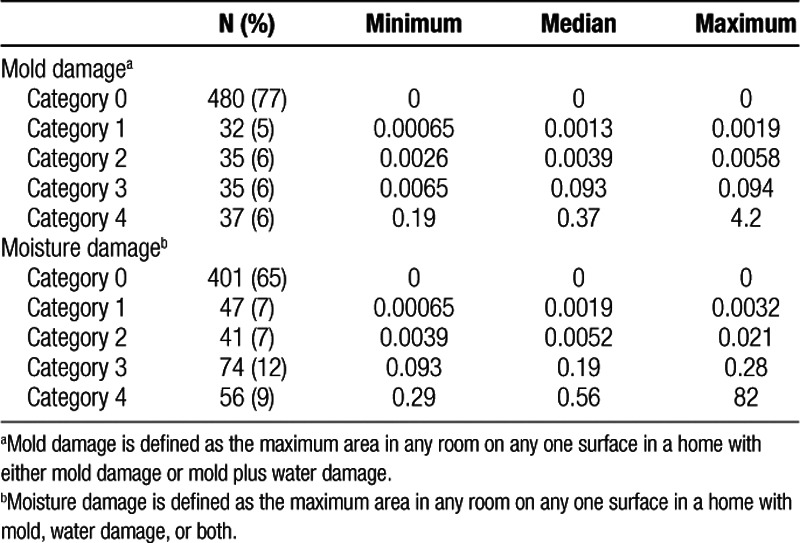
Categorical moisture and mold damage minimum, median, and maximum (m^2^).

### Wheeze at age 3

Results of univariate analyses of the association between the health outcomes and each exposure variable are presented in eTable 2; http://links.lww.com/EE/A93. In unadjusted models, the highest categories of mold damage (≥0.19 m^2^) and moisture damage (≥0.29 m^2^) were significantly associated with increased age 3 wheeze (odds ratios [OR] = 3.64; 95% confidence interval [CI] = 1.64, 7.79 and OR = 2.34; CI = 1.15, 4.57, respectively). Continuous variables for both mold damage area and moisture damage area were significantly associated with age 3 wheeze (OR = 1.83; CI = 1.10, 3.01 and OR = 1.67; CI = 1.05, 2.65, respectively). No significant associations were observed between moldy odor and wheezing at age 3.

**Figure 4.**
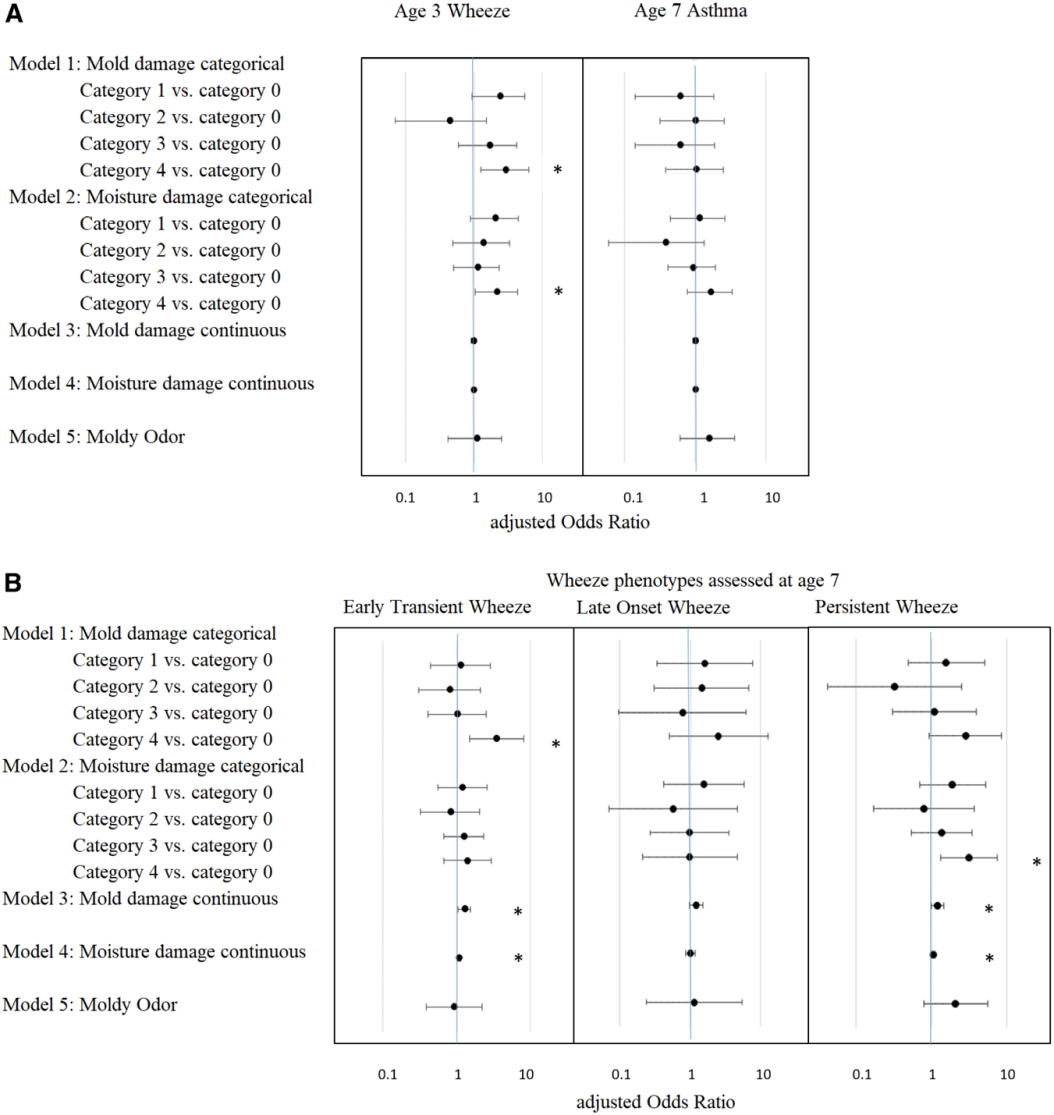
Adjusted odds ratios of having wheeze or asthma by exposure variables. A, Age 3 wheeze and age 7 asthma. B, Age 7 wheeze phenotypes divided into early transient, late onset, and persistent wheeze. Error bars demonstrate 95% confidence interval. Odds ratio adjusted for neighborhood socioeconomic status, income, presence of cockroaches, rats, and mice. *95% confidence interval >1 or <1.

The adjusted odds ratios (aOR) and CI from five final models are presented in Figure 4. Several significant associations were found between the exposure variables and age 3 wheeze. In our analysis of categorical mold damage, the highest category of mold (≥0.19 m^2^) was significantly associated (aOR = 2.91; CI = 1.27, 6.43) with wheezing at age 3. Similarly, the highest category of moisture damage (≥0.29 m^2^) was significantly associated (aOR = 2.16; CI = 1.03, 4.33) with wheeze at age 3. We did not observe a significant relationship between mold damage or moisture damage when these were treated as continuous exposures. Similarly, no significant associations were observed between moldy odor and wheezing at age 3.

### Wheeze at age 7

In univariate analyses of wheeze phenotypes at age 7, mold damage ≥0.19 m^2^ was associated with significantly increased ET and PS wheeze (OR = 4.08; CI = 1.77, 9.38 and OR = 4.08; CI = 1.41, 11.84, respectively). In addition, moisture damage greater than ≥0.29 m^2^ (OR = 3.79; CI = 1.68, 8.58), continuously modeled moisture damage (OR = 1.91; CI = 1.07, 3.40), and moldy smell (OR = 2.30; CI = 1.04, 5.12) were also associated with significantly increased PS wheeze.

In adjusted models, moisture damage ≥0.29 m^2^ remained significantly associated with PS wheeze (aOR = 3.18; CI = 1.34, 7.53). Mold damage ≥0.19 m^2^ had a marginally significant association with PS wheeze (aOR = 2.83; CI = 0.94, 8.56) and was significantly associated with ET wheeze at age 7 (aOR = 3.48; CI = 1.48, 8.18) (Figure 4). Persistent wheeze was also significantly associated with continuously modeled variables for both mold damage and moisture damage (aOR = 1.21; CI = 1.00, 1.47 and aOR = 1.07; CI = 1.00, 1.13, respectively). Continuously modeled mold damage and moisture damage were also both associated with significantly increased ET wheeze (aOR = 1.26; CI = 1.05, 1.52 and aOR = 1.07; CI = 1.00, 1.13, respectively). Moldy smell was not determined to be significantly associated with wheeze at age 7; however, there was a marginally significant association with PS wheeze (aOR = 2.10; CI = 0.79, 5.61). No significant associations, in unadjusted or adjusted models, were found with any of the exposure variables for LO wheeze.

### Asthma at age 7

In unadjusted analyses, no significant associations were found with any of the exposure variables for asthma. Moisture damage ≥0.29 m^2^ had a marginally significant association with OR = 1.99, CI = 0.96, 3.91. In adjusted analyses, no significant associations were found.

## Discussion

Wheezing at age 3 and ET and PS wheezing at age 7 were found to be associated with mold and moisture damage in the child’s home at age 1. The strongest associations were seen between the highest categories of damage and the wheezing outcomes.

We found significant associations of persistent wheeze with both moisture and mold damage areas. Every additional 1 m^2^ of mold damage was associated with odds increased 21% for PS wheeze and 26% for ET wheeze, and every additional 1 m^2^ of moisture damage was associated with odds increased by 7% for both PS and ET wheeze. With moisture damage greater than 0.29 m^2^, compared with no moisture damage, the odds for PS wheeze increased 218% (aOR = 3.18), and with mold damage greater than 0.19 m^2^, compared with no mold damage, the odds of ET wheeze increased 248% (aOR = 3.48). Karvonen et al^10^ had found that wheezing apart from a cold, with subjects contributing up to 7 repeated observations, was significantly associated with major moisture damage in the kitchen (aOR = 2.20; CI = 1.08, 4.49).^10^ Karvonen et al’s^10^ definition of wheezing and major moisture damage is similar to our definition of PS wheeze and our upper category of moisture damage; both studies yielded similar statistically significant aORs.

We found that the odds of age 3 wheeze almost tripled (aOR = 2.9) when the home had high levels of mold (≥0.19 m^2^) compared with no mold and almost doubled (aOR = 2.2) when the home had high levels of moisture damage (≥0.29 m^2^). The finding on mold damage is consistent with our previous findings. Iossifova et al^21^ determined infants’ odds for wheeze almost quadrupled (aOR = 4.4) if high visible mold (>0.2 m^2^) was present in the home. Furthermore, at age 3, these children’s odds were six times as high (aOR = 6) for having wheezing with atopy relative to those with no visible mold.^12^

In our previous analyses, we categorized observed mold as high, if the damaged area was >0.2 m^2^.^11^ This categorization was not data-driven but was based a priori on mold cleanup guidelines.^22^ The highest category for mold damage (≥0.19 m^2^) set in this study by quartile boundaries of nonzero values provided a similar limit, as did the highest category for moisture damage of ≥0.29 m^2^ (i.e., damage from water damage, mold, or both). The categorical variable findings suggested there may be a specific threshold associated with increased risk of wheezing or asthma and the threshold could possibly occur between the third and fourth quartile (0.0065–0.19 m^2^ for mold damage and 0.093–0.29 m^2^ for moisture damage); however, this study did not specifically determine where or if such thresholds occur. If there were thresholds in these relationships, as potentially indicated, then continuous exposure models may not be the best method for estimating the relationships between these health effects and exposure. While the highest categories used in these analyses (>0.19 m^2^ mold damage and >0.29 m^2^ moisture damage) were associated with significantly and substantially increased wheezing, we would not suggest using these values as health-relevant thresholds. The data below these levels were too sparse to assess the shape of the relationship or to explore potential health-relevant thresholds. Research including more data below these levels is needed to explore the shapes of the exposure–response relationships, to identify any truly health-relevant thresholds.

Age 7 asthma was not found to be associated with mold or moisture damage, moldy odor, or water damage history. Of those individuals that completed the asthma test, 72% of the PS wheeze phenotype also had asthma, and we did find a significant association of PS wheeze with mold and moisture damage. While differing definitions have been used in studies for asthma and wheezing, persistent wheeze has been previously shown to be associated with long-term deficits in lung function.^23–26^ Oksel et al^27^ found that all wheeze phenotypes had significantly diminished lung function in school-age children, and the association with asthma was strongest for persistent wheeze.^28^ In addition, the authors explored the idea that early-life episodic wheeze may not be a harmless prognosis for some with transient wheeze.^27^ One reason asthma was not found to be significantly associated with mold or moisture damage could be the strict definition of asthma used in the analyses. As 25 participants who were likely to be asthmatic were unable to complete the required test for asthma diagnosis, this reduced both the number of asthmatics and the overall number of participants available for analyses. This limited our ability to identify significant associations of age 7 asthma with mold or moisture damage, moldy odor, or water damage history. Using post-hoc sample calculations, we have determined that an additional 25 asthmatics would increase the statistical power 63%–72%. To achieve a statistical power of 80%, 55 additional asthmatics would have been needed. Potentially, with these added participants, the confidence interval for the highest quartile of moisture damage or moldy odor might have been reduced and a significant aOR produced.

Several studies reported that history of water damage and/or moldy smell was associated with increased risk of developing wheeze,^6,29–31^ while other studies have not found significant associations.^10,32,33^ In our study, history of water damage was not determined to be significantly associated with outcomes in the univariate models. Our adjusted models for moldy odor, intended to identify causal links, were adjusted for mold area. In these models, moldy odor was not significantly associated with increased risk of wheezing or asthma. Without the adjustment, however, moldy odor was significantly associated with PS wheeze, demonstrating that moldy odor may still be a useful indicator of dampness and mold-related risk even if it is not a direct causal factor of health effects. Metrics including water damage, visible mold, and moldy odor have been previously associated with increased health risks; however, the mechanisms behind these associations remain unclear.^11,21,31^

The third National Health and Nutrition Examination Surveys (NHANES III) determined 54.3% of the US population had positive test responses to one or more allergens^13,34^; therefore, our findings are generalizable to the US population, as our selected study population had at least one parent with allergic sensitization. A limitation of this study was the sparse amount of data in the lower exposure groups. The limited data did not allow for a more detailed analysis of the lower exposure levels. In addition, the exposures were collected at age 1, to evaluate early-life exposure and resulting health outcomes during early adolescence. However, later exposure assessments were not included in this analysis, preventing consideration of exposures changing over time. Another possible limitation involved the measurements of the mold and water damage. Mold and water damage typically have irregular, asymmetrical shapes, and while the researchers were trained to record the information in a consistent and standardized way,^11^ subjective estimations by multiple researchers were likely to have introduced variability into these data. We assume the resulting nondifferential misclassification would have resulted in underestimation of any true associations. In addition, selected homes (5%) had a subsequent home visit within 2 months for quality control to ensure the reliability of the home characteristics data.^11^

## Conclusions

Ideally, methods to quantify the relevant exposures to microorganisms and/or their products are needed to estimate the health risks in damp or moldy buildings, so that health-relevant guidelines can be set; however, this has not been possible using current microbiological measurement methods.^35^ The goal of this study was to explore, as practical and currently feasible alternatives, the use of quantified measurements of visible mold and moisture damage, in conjunction with qualitative information on moldy odor and history of water damage, to characterize exposure–response relationships of dampness and mold with respiratory health outcomes. The highest categories of both quantified variables, moisture damage (≥0.29 m^2^) and mold damage (≥0.19 m^2^), had significant associations with negative health outcomes for age 3 wheeze and age 7 ET and PS wheeze. Future studies should include more exposure data below these levels to assess the shape of the relationship and explore potential health-relevant thresholds. In addition to moisture and mold damage, moldy smell may be an indicator of the health risks associated with dampness and mold.

## Conflicts of interest statement

The authors declare that they have no conflicts of interest with regard to the content of this report.

## Supplementary Material


