# Supplementary material for: Quantitative and semiquantitative estimates of mold exposure in infancy and childhood respiratory health
Source: Environ Epidemiol. 2020 Jun 19;4(4):e101. doi: 10.1097/EE9.0000000000000101 (PMC7423528; doi:10.1097/EE9.0000000000000101)

**Supplemental Digital Content**

**Quantitative and semi-quantitative estimates of mold exposure in infancy and childhood respiratory health**

*Jennie Cox^1^, Patrick Ryan^1,2^, Jeff Burkle^2^, Roman Jandarov^1^, Mark J. Mendell^3^, Gurjit Khurana Hershey^4^, Grace LeMasters^1^, Tiina Reponen^1^*

*^1^Department of Environmental Health, University of Cincinnati, P.O. Box 670056, Cincinnati, OH, USA ^2^Division of Biostatistics and Epidemiology, Department of Pediatrics, Cincinnati Children's Hospital Medical Center, Cincinnati, OH, USA, ^3^Indoor Epidemiology, El Cerrito, CA, USA, ^4^Division of Allergy and Immunology, Cincinnati Children’s Hospital Medical Center, Cincinnati, OH, USA.*

Contact name: Dr. Jennie Cox

Contact phone: 513-558-0507

Contact email: roejd@ucmail.uc.edu

Running head: Observed mold and respiratory health

Conflicts of Interest: The authors declare no conflicts of interest

Sources of Funding: This study was supported by US Housing and Department (Grant OHHHU00237-17). Additional funding was provided by the National Institute for Occupational Safety and Health through the University of Cincinnati Education and Research Center Grant #T42OH008432 and the National Institute of Environmental Health Sciences grant R01ES11170.

Data will be available from the corresponding author upon request


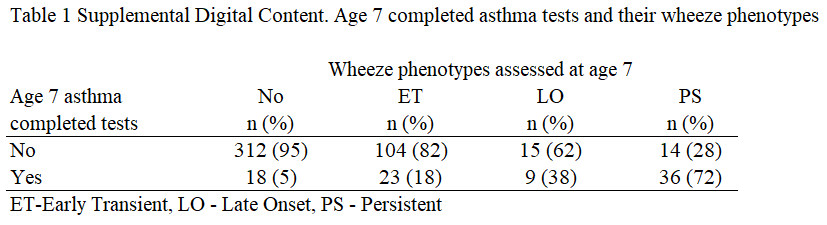


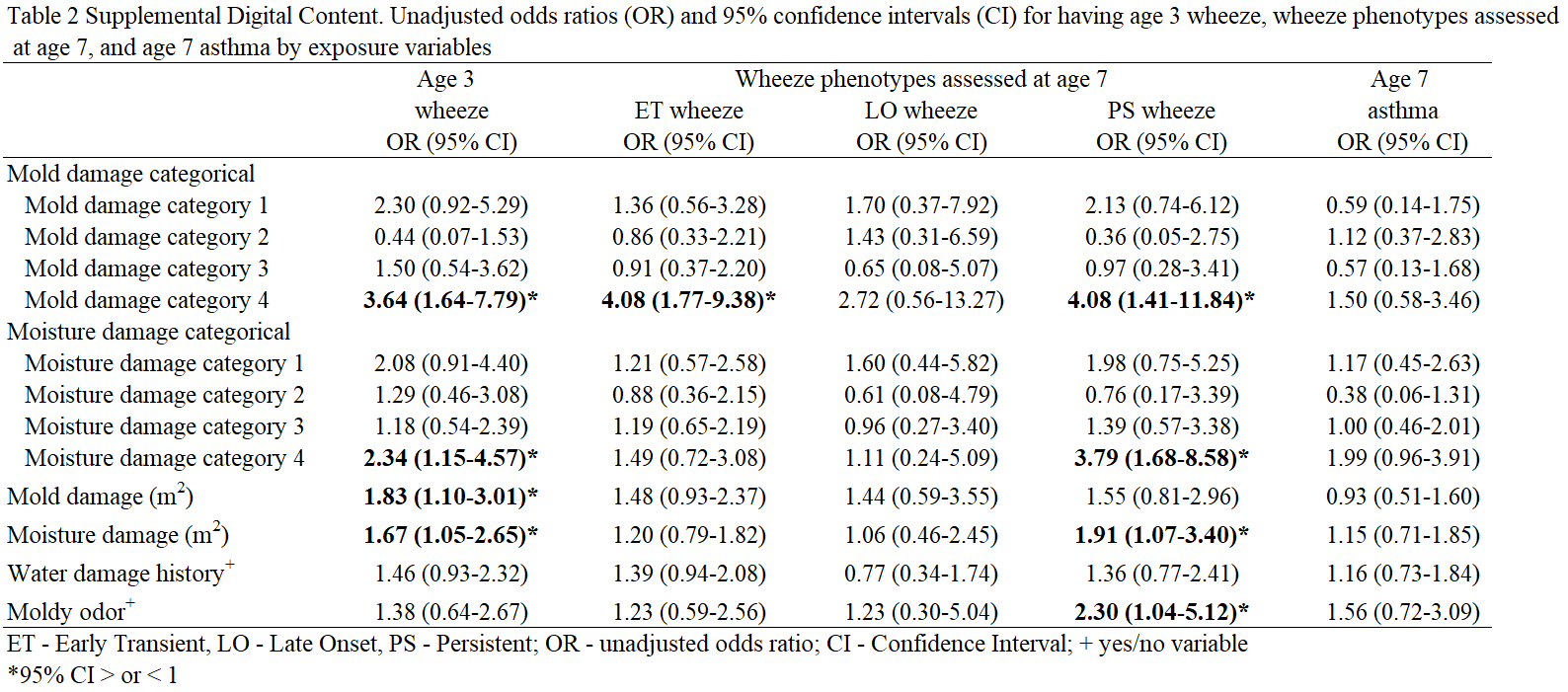


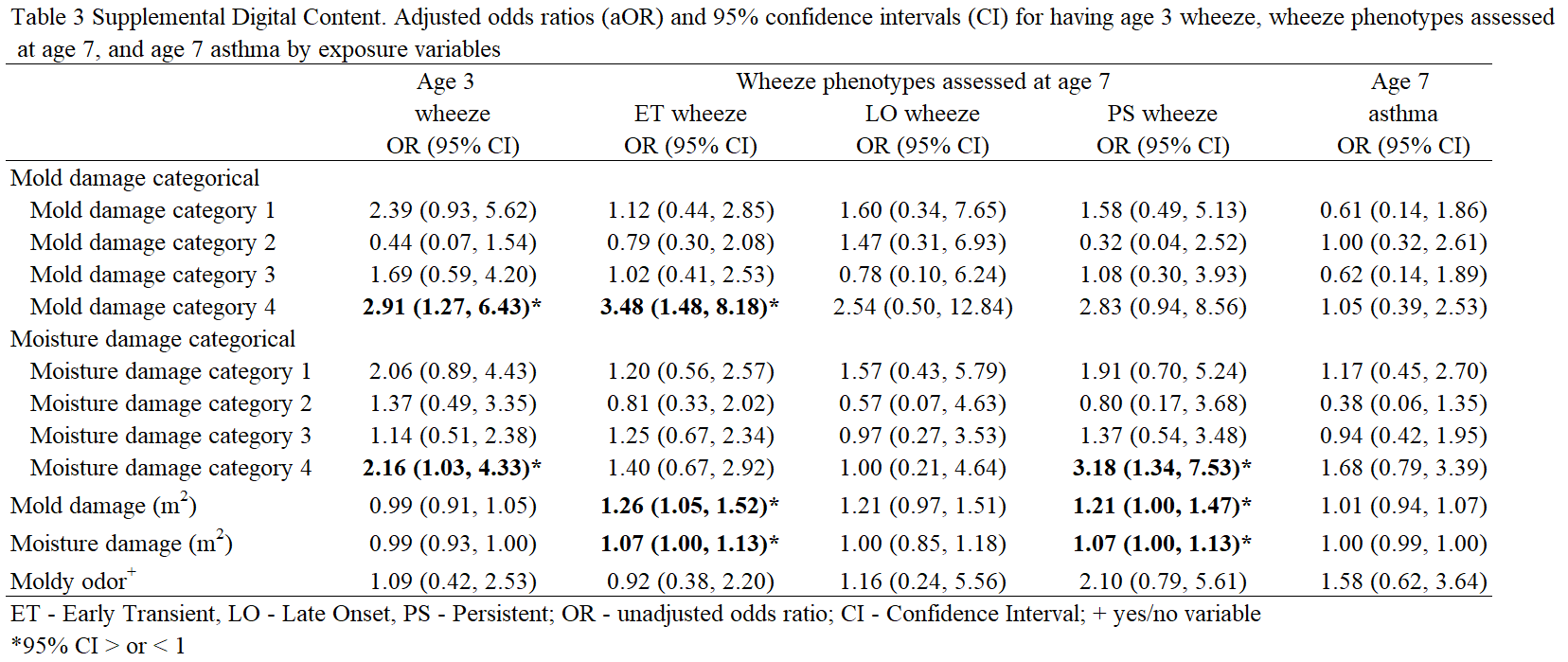

Supplement: Supplementary file 1 [file ee9-4-e0101-s001.docx]
